# The Role of MicroRNAs in Breast Cancer Migration, Invasion and Metastasis

**DOI:** 10.3390/ijms131013414

**Published:** 2012-10-18

**Authors:** Joy Tang, Aamir Ahmad, Fazlul H. Sarkar

**Affiliations:** 1 Department of Pathology, Wayne State University School of Medicine and Karmanos Cancer Institute, Detroit, MI 48201, USA; E-Mails: ec1265@wayne.edu (J.T.); ahmada@karmanos.org (A.A.); 2 Department of Oncology, Wayne State University School of Medicine and Karmanos Cancer Institute, Detroit, MI 48201, USA

**Keywords:** miRNAs, breast cancer, metastasis, invasion, tumor suppressor, oncogenic

## Abstract

MicroRNAs (miRNAs) are a major class of small, noncoding RNA molecules that regulate gene expression by targeting mRNAs to trigger either translational repression or mRNA degradation. They have recently been more widely investigated due to their potential role as targets for cancer therapy. Many miRNAs have been implicated in several human cancers, including breast cancer. miRNAs are known to regulate cell cycle and development, and thus may serve as useful targets for exploration in anticancer therapeutics. The link between altered miRNA signatures and breast cancer development and metastasis can be observed either through the loss of tumor suppressor miRNAs, such as let-7s, miR-30a/31/34a/125s/200s/203/205/206/342 or the overexpression of oncogenic miRNAs, such as miR-10b/21/135a/155/221/222/224/373/520c in breast cancer cells. Some of these miRNAs have also been validated in tumor specimens of breast cancer patients, underscoring their potential roles in diagnostics, as well as targets for novel therapeutics for breast cancer. In this review article, we will provide an overview and update of our current understanding of the mode of action of several of these well characterized miRNAs in breast cancer models. Therefore, better understanding of the gene networks orchestrated by these miRNAs may help exploit the full potential of miRNAs in regards to cancer diagnosis, treatment, and therapeutics.

## 1. Introduction

MicroRNAs (miRNAs) are tiny, regulatory RNA molecules, approximately 18–25 nucleotides in length that post-transcriptionally modulate gene expression [1–3]. They regulate genes by binding to site(s) within the 3′ untranslated region (UTR) of multiple target messenger RNAs (mRNAs), which results in either translational repression or degradation of mRNAs [4–6]. Although miRNAs were first identified in the early 1990s, it is only in the past decade that their potential has been more widely explored. Recent profiling studies have identified miRNAs that are aberrantly expressed in human cancers, and the functions of some such miRNAs in breast cancer have been investigated [7]. miRNAs are now widely believed to play an essential role in many malignancies, acting as either tumor suppressors or oncogenes [3,7,8]. Target genes are regulated by miRNAs through binding to sequence complementarities, either perfectly or imperfectly. The majority of miRNA function is based on repression of their target genes, which means that a certain miRNA will be tumor suppressive if its target gene is an oncogene [8,9]. Gene regulation by miRNAs is important for the onset and progression of several human cancers [3,10–12].

**Figure 1:**
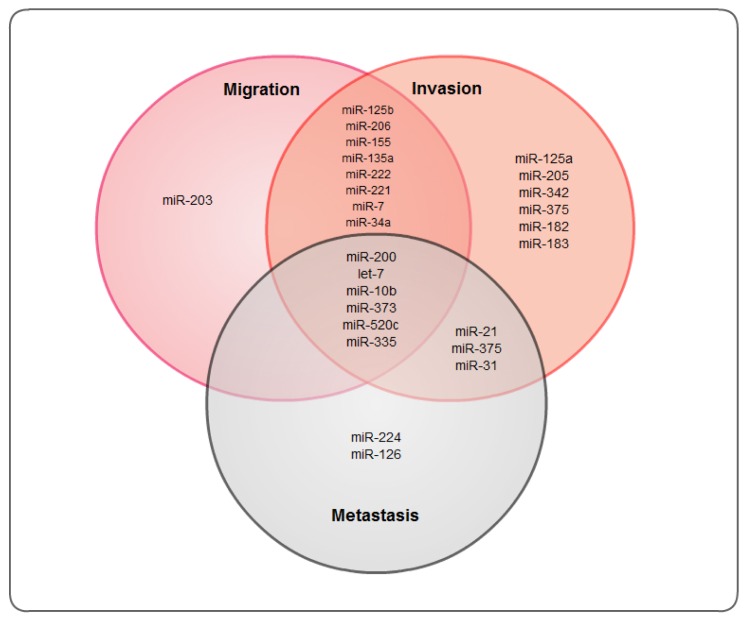
Venn diagram showing an overlapping role of several miRNAs in breast cancer migration, invasion and metastasis.

Better understanding of miRNAs can be especially useful in improvements for breast cancer treatment. Breast cancer is one of the leading causes of cancer-related deaths and a common leading malignancy among women in the United States [13–15]. Many recent studies on breast cancer have analyzed various miRNAs that might influence breast cancer progression and development [12,16,17] (Figure 1). Although some target genes and miRNA profiles have been identified, the mechanistic overview and full potential of miRNAs is still not realized. Further studies will improve the utilization of miRNAs in targeted therapeutics.

## 2. The Role of miRNAs in Breast Cancer

MicroRNAs function in various temporal and tissue-specific eukaryotic gene regulation, and thus they can potentially target any mRNAs in humans. The miRNAs are involved in a majority of biological processes, including cell cycle regulation, cell growth, apoptosis, cell differentiation and stress response [18,19]. Further studies have shown that there is a connection between miRNA function and several diseases, including cancer. More specifically, miRNAs can either modulate oncogenic or tumor suppressor pathways, or their expression can be regulated by oncogenes or tumor suppressor genes [20,21]. This may be because more than 50% of miRNA genes are located at chromosomal regions, including fragile sites and regions of deletion and amplification that are genetically altered in human cancers [22,23]. Deeper comprehension of miRNA activity in the human body can lead to promising new therapies for the management of human malignancies, including breast cancer.

Currently, it is estimated that, on average, one in eight women will develop breast cancer in her lifetime [24]. Although progress has been made on breast cancer diagnosis and treatment, there are still many unexplored areas, particularly in cancer therapeutics. Various studies suggest that miRNAs play a critical role in breast cancer [2,25–31]. Over the past few years, miRNAs have been implicated in cell proliferation, invasion, angiogenesis, and metastasis of breast cancer cells [3]. Since cancer malignancies can develop from either a reduction or deletion of a tumor suppressor miRNA or from amplification or overexpression of an oncogenic miRNA, breast cancer metastasis can also be enhanced by increased expression of prometastatic or downregulation of anti-metastatic miRNAs [7]. These results suggest that there is a potential role for miRNAs not only to facilitate in the diagnosis, prognosis and predictions in treatment responses, but also to function as novel therapeutic targets and replacement therapies. Below, several miRNAs and their functions as tumor suppressors or oncogenes in human breast cancer are discussed.

## 3. Tumor Suppressor miRNAs

### 3.1. miRNA-200 Family

The miR-200 family is comprised of five members: miR-200a, miR-200b, miR-200c, miR-141 and miR-429. These members are then divided into two clusters based on chromosomal location, with cluster one consisting of miR-200b, miR-200a, and miR-429, located on chromosome 1 in humans, and cluster two consisting of miR-200c and miR-141, located on chromosome 12 in humans [3]. The gene sequence between the two clusters only differs by a single nucleotide, which suggests that the members of miR-200 can potentially target a common subset of genes for similar physiological effects [32,33]. Due to the relatively high degree of overlap in target genes between the two clusters, all members of the miR-200 family are good candidates for targeting common subsets of genes, such as those that are involved in breast cancer metastasis [34,35].

Metastasis and cell invasion are both characteristic of malignant tumor progression [36]. Further research shows that the acquisition of epithelial-to-mesenchymal transition (EMT) typically results in invasion and metastasis by promoting cell detachment, which thereby increases tumor cell mobility [6]. EMT also contributes to the malignant transformation of many human cancers. The loss of epithelial differentiation and transition to mesenchymal phenotype is linked to progression of most cancer malignancies. Epithelial cells typically have normal cell-to-cell junction and adhesion, while mesenchymal cells have weaker cell wall adhesion, making them more motile and likely to enhance invasive characteristics. EMT has been found to play an essential role in tumor invasion, metastatic dissemination, and the acquisition of resistance to current cancer therapies [37]. It is activated by EMT-inducing transcriptional repressors, which include members of the Snail family and the ZFH family of transcription factors [7,38]. Emerging studies indicate that the miRNA-200 family could regulate the EMT process by targeting specific molecular markers of EMT [12].

The miR-200 family targets specific molecular markers of EMT, including E-cadherin, which is the marker for epithelial phenotype, vimentin, and ZEB1 and ZEB2, which are the markers of mesenchymal phenotype [3,39]. Furthermore, ZEB1 has been shown to be an essential EMT activator in human cancers, including breast cancer, and promoted metastasis of tumor cells in mouse models [7,40,41]. The miR-200 family has been shown to induce epithelial phenotype by suppressing the expression of EMT inducers, ZEB1 and ZEB2 [42,43]. In a pioneering work by Gregory *et al*. [43], it was suggested that the entire miR-200 family is downregulated upon exposure to transforming growth factor-β (TGF-β). TGF-β is a cytokine that is known to induce the EMT phenotype [37,44,45]. Reexpression of the miR-200 family significantly inhibited EMT that was induced by TGF-β, while inhibition of the miR-200 family resulted in the induction of EMT phenotype. Furthermore, there were increased levels of ZEB1 and ZEB2 following the induction of EMT, which suggests that the miR-200 family is a negative regulator of the mesenchymal markers, ZEB1 and ZEB2. Another study [46] that focused on miRNAs suppressed by ZEB1 also showed that the most affected miRNAs were members of the miR-200 family. It was revealed that ZEB1 can bind to highly conserved sites in the promoter and directly suppress transcription of an entire cluster of the miR-200 family. Furthermore, ZEB1 is also a target of miR-200c, which indicates that there is an EMT-inducing feed-forward cycle.

A recent study on miR-200 revealed that three miRNA clusters, miR-200c-141, miR-200b-200a-429, and miR-183-96-182, are all involved in the regulation of self-renewal in cancer stem cells (CSCs) and normal stem cells [47]. The three miRNA clusters are downregulated in human breast CSCs, normal human cells, and mouse mammary cells. Emerging evidence has demonstrated that the *BMI1* gene, which promotes stem cell self-renewal, was directly inhibited by these miRNA clusters. In addition, ectopic overexpression of miR-200c in embryonal carcinoma cells resulted in neural differentiation and suppressed tumorigenicity of breast CSCs *in vivo* [48]. Our own investigations into the mechanisms of phosphoglucose isomerase (PGI)/autocrine motility factor (AMF)-induced metastases of breast cancer cells *in vitro* as well as *in vivo* have suggested the involvement of miR-200s [12]. We found that the PGI/AMF-induced EMT is marked by induced E-cadherin expression and reduced vimentin/ZEB1/ZEB2 expression. Furthermore, we observed a mechanistic involvement of miR-200s in this regulation of EMT by PGI/AMF, where overexpression of miR-200s was found to reverse the PGI/AMF-induced EMT and, conversely, silencing of miR-200s was found to induce EMT even in the PGI/AMF-silenced breast cancer cells. We further confirmed our results *in vivo* in an experimental pulmonary metastases model where PGI/AMF or silencing of miR-200s induced lung metastases and downregulation of PGI/AMF or overexpression of miR-200s significantly reduced these lung metastases. Thus, evidence from all these studies suggests that the miR-200 family plays a crucial role in regulation of breast cancer metastasis and aggressiveness.

### 3.2. miRNA-125

The miR-125 has two known isoforms in humans: miR-125a and miR-125b. Altered expression of miR-125 has been observed in several malignancies, including breast cancer [49,50]. The miR-125a and miR-125b were both found to be significantly downregulated in breast cancer patients. Guo *et al.* [51] explored the role of miRNA-125 in breast cancer after observing [22,52] that there were decreased levels of miRNA-125 in breast tumors in comparison to normal breast tissues. Their work [51] showed that miRNA-125a is inversely correlated with HuR expression in various breast carcinoma cell lines. HuR is an RNA binding protein (RBP) that is upregulated in several different cancers. The miR-125a represses translation of HuR through a target site in the 3′ UTR. Overexpressing miR-125a led to decreased HuR protein levels, suppressed cell growth, and reduced cell migration and proliferation. These results suggest that miR-125a can potentially aid in tumor suppression in breast cancer by utilizing HuR as a direct and functional target.

Further research showed that miR-125a and miR-125b are both downregulated in HER2-overexpressing breast cancers [53]. HER2, the human epidermal growth factor receptor 2, has no ligand-binding domain and thus cannot bind growth factors. It binds to other ligand-bound epidermal growth factor receptor family members to create a heterodimer [7]. Amplification of HER2 is common in numerous cancer patients, including those with breast tumors. The miR-125a and miR-125b function as tumor suppressors in SKBR3 cells, a HER2-overexpressing human breast cancer cell line, by suppressing *HER2* mRNA and protein levels. This results in reduced cell growth, motility and invasiveness [54]. The c-Raf has also been proposed as a target of miR-125b leading to its antiproliferative effect [55].

### 3.3. Let-7 Family

Lethal-7 (let-7), one of the first mammalian miRNAs to be identified, was initially discovered to play a role in the developmental timing of *Caenorhabditis elegans* and is conserved throughout the animal phyla [4,7,56]. The let-7 family consists of 12 members: let-7-a1, let-7-a2, let-7-a3, let-7-b, let-7-c, let-7-d, let-7-e, let-7-f1, let-7-f2, let-7-g, let-7-i and miR-98 [3,57]. Studies have shown that let-7 is often absent in cells that are less differentiated, exhibit a mesenchymal phenotype and represent more advanced cancer [58]. Thus, in accordance, higher levels of let-7 are expressed in cells that are more differentiated, exhibit an epithelial phenotype and represent less advanced cancer. An *in vivo* study of let-7 showed that its administration was effective against lung and breast cancers in mice and further computational analysis suggested that let-7 would be effective in estrogen receptor positive (ER+) metastatic breast cancer [6]. let-7 also regulates apoptosis and CSCs differentiation, which suggests that it has high potential of being utilized in cancer therapy [59].

An earlier study investigated the expression of let-7 in breast cancer cells and demonstrated that let-7-fl is expressed in multiple breast cancer cell lines [60]. Further research investigated the role of let-7 in distinguishing breast tumor-initiating cells (BT-IC) from other differentiated progeny. There was reduced expression of let-7 in BT-IC, but the level of let-7 increased with differentiation [61]. Administration of let-7 in BT-IC also resulted in reduced proliferation and reduced metastasis *in vivo*. On the other hand, when let-7 was silenced, non-T-IC experienced higher self-renewal ability. The research suggests that let-7 affects self-renewal ability of cancer cells, so cancer therapy by targeting let-7 in breast cancer patients could be beneficial and could serve as a novel treatment option.

Dangi-Garimiella *et al*. proposed a role for let-7 in the metastasis of breast cancer. Their study showed that signaling through let-7 regulates the repressive action of Raf kinase inhibitory protein (RKIP) on metastasis of breast cancer cells [62]. RKIP is a suppressor gene in human breast cancers that inhibits MAPK, G protein-coupled receptor kinase-2 and NF-κB signaling cascades [63,64]. Its expression is thus expected to be reduced in invasive cell lines. RKIP, in accordance, inhibited invasion by metastatic breast cancer cells and repressed breast tumor cell intravasation in mouse models. Inhibition of MAPK resulted in decreased transcription of *LIN28*, which led to the downregulation of let-7 targets. The let-7 targets are mechanistically linked to metastasis of carcinomas, and thereby their downregulation enhances the process by which RKIP exerts its tumor suppressor abilities [3,65]. Elevated levels of let-7 family members results in the downregulation of its targets, such as HMGA2, which are mechanistically linked to metastasis of cancer cells.

Another proposed role for let-7 is its involvement in signaling through estrogen receptor. Previous research revealed that estradiol (E2) may encourage increased levels of eight members of the let-7 family [66]. E2 also acts as a ligand for both estrogen receptor alpha (ER-alpha) and estrogen receptor beta (ER-beta). Recent analysis reported that there is an inverse association between the expression of ER-alpha and various members of the let-7 family [67]. The let-7 family miRNAs were found to be downregulated in human breast cancer cells at stages of ductal carcinoma *in situ* (DCIS) and invasive ductal carcinoma (IDC) in comparison to the benign stage. Overexpression of let-7 family members in ER-positive cells resulted in downregulated ER-alpha activity, reduced cell proliferation and induced apoptosis. Furthermore, it was found that let-7 family members target similar genes and have similar functions.

Studies surrounding let-7 suggest that it can be used as a future cancer therapeutic. It is now widely accepted that let-7 levels are high in normal cells and reduced in invasive cancer cell lines. However, the mechanism of let-7 deregulation and its role in tumorigenesis is currently not fully understood. Further studies on the molecular mechanisms behind let-7 activity in cancer would improve treatment plans for therapeutic applications.

### 3.4. miRNA-205

The miR-205 has been implicated as a tumor suppressor in breast cancer [68]. The miR-205 expression is reduced in breast cancer tissue samples in comparison to normal breast tissue. Furthermore, its expression is limited to myoepithelial cells and is reduced in matching tumor samples, which indicates that it is associated with a good prognosis. The miR-205 was shown to negatively modulate ZEB1 and is thereby negatively linked to the acquisition of EMT [43,69]. This suggests that miR-205 activity is very similar to that of the miR-200 family, which is also associated with negative regulation of EMT. Further support for miR-205’s potential to identify breast tumors from normal tissue revealed reduced expression in breast cancer cell lines MCF-7 and MDA-MB-231 in comparison to normal MCF-10A breast epithelial cells. In addition, transfecting miR-205 in cancer cells resulted in the inhibition of cell proliferation, clonogenicity and invasion [68]. The downregulation of miR-205 in metastatic breast cancer cell lines suggests that it is an ideal therapeutic target in cancer treatment.

Another study explored miR-205 target genes in breast cancer patients. It revealed that miR-205 directly targets HER3, a receptor tyrosine kinase of the epidermal growth factor receptor (EGFR) family [70]. Members of EGFR are typically involved in tumorigenesis when their signaling functions are deregulated [71]. The miR-205 inhibits HER3, inactivating the downstream mediator Akt, which results in the suppression of the PI3K/Akt signaling pathway. In breast cancer, overexpression of HER2 is characteristic of an aggressive subset of tumors [72]. HER2 activity is dependent on the transinteraction with other HER family members. In particular, its relationship with HER3 leads to the activation of the PI3K/Akt pathway, progression of tumor and resistance to chemotherapy [73]. HER3 thus plays a critical role in HER2-mediated tumorigenesis. The miR-205 can contribute to cancer therapeutics due to its negative regulation of the HER3 receptor, which affects survival and proliferation of breast cancer cells. The miR-205 can inhibit breast cancer cell proliferation and improve response to specific targeted therapies as well.

### 3.5. miRNA-206

The miR-206 has been shown to suppress breast cancer migration [74,75]. It was first discovered by using miRNA microarrays to compare miRNA expression between normal and breast cancer cells [22]. The miR-206 is upregulated in estrogen receptor negative (ER−) breast cancers and also inhibits the expression of the estrogen receptor gene *ERα* (*ESR1*) through two binding sites in the *ESR1* 3′ UTR [76]. Other work also showed that miR-206 expression was reduced in ERα-positive human breast cancer tissues and that miR-206 suppresses ESR1 expression in addition to inhibiting the growth of MCF-7 breast cancer cells [77]. The miR-206 was shown to induce cell cycle arrest and inhibit estrogen-induced proliferation. The tumor suppressive role of miR-206 was further confirmed in another study that demonstrated miR-206 to be downregulated in metastatic breast cancer cells in comparison to normal parental cells [78]. Reexpression of miR-206 resulted in reduced invasive capacity and altered cell morphology, which can also limit the migration of metastatic cells. These findings suggest a potential role for miR-206 in breast cancer therapy.

### 3.6. miRNA-34a

The miR-34a is a transcriptionally regulated miRNA by the p53 network, which has been shown to be downregulated in multiple cancers [52]. A previous study has demonstrated that in breast cancer, miR-34a levels are lower in triple negative and mesenchymal-type breast cancer cell lines when compared to the normal epithelial cell lines [79]. The study proposed that p53 mutations may contribute to lower miR-34a expression. In terms of investigating the miRNA’s sensitivity to radiation, the authors compared the sensitivity of the normal breast epithelial cell line HMEC, the HER-2+ cell line UACC-812, and the mesenchymal-type cell line MDA-MB-231. Results have indicated that MDA-MB-231 cells, which have low miR-34a levels, showed increased sensitivity to radiation in comparison to HMEC and UACC-812, which both express high levels of miR-34a [79]. In addition, MDA-MB-231 cells were protected from radiation-induced death if miR-34a levels were increased. In accordance, downregulating miR-34a levels would enhance radiation-induced deaths. The results of this study suggest that miR-34a is essential for MDA-MB-231 cell survival when considering radiation sensitivity.

Another study examined the effect of miR-34a on breast cancer development and found that its levels are downregulated in five different breast cancer cell lines in comparison to the normal epithelial cell line 184A1 [80]. Ectopic expression of miR-34a levels in breast cancer cells also led to reduced cell proliferation, invasion, and induced apoptosis. It was also found that miR-34a targets, Bcl-2 and SIRT1, are in reverse correlation with ectopic expression of miR-34a. The study concluded that miR-34a is able to inhibit breast cancer cell proliferation and migration through downregulation of Bcl-2 and SIRT1. Since manipulating miR-34a levels affects radiation sensitivity in breast cancer cells, miR-34a could have great potential for breast cancer therapy.

### 3.7. miRNA-31

The miR-31, which is known to have pleiotropic effects on breast cancer metastasis, has been shown to inhibit metastasis at multiple steps by inhibiting the expression of prometastatic genes [81]. Normal levels are exhibited in normal breast epithelial cells, but miR-31 is almost undetectable in metastatic breast cancer cells *in vivo*. It is also expressed in decreased levels in non-metastatic breast cancer cell lines. Investigations that explored miR-31 expression in MDA-MB-231 and SUM-159 breast cancer cells found that ectopic expression of miR-31 *in vivo* and *in vitro* hindered invasion and metastatic colonization [81]. The experiment showed that even though overexpressing miR-31 in breast cancer cells resulted in larger tumors and increased proliferation, the cancers were encapsulated and less invasive. Furthermore, miR-31 also reduces cell survival and the ability to form secondary tumors. In accordance, inhibiting miR-31 resulted in increased invasiveness and metastasis *in vivo*.

The miR-31 may regulate at least 200 different mRNAs in mammalian cells. At least six targets have been identified, which include frizzled3 (*Fzd3*), integrin α-5 (*ITGA5*), myosin phosphatase-Rho interacting protein (*M-RIP*), matrix metallopeptidase 16 (*MMP16*), radixin (*RDX*) and *RhoA* [82]. In order to investigate whether miR-31 regulation of these target genes affects metastatic function, the six identified target genes were reexpressed in miR-31-expressing cells. Results showed that only *ITGA5*, *RDX*, and *RhoA* could reverse the motility defects and impaired invasion, which suggests that these three genes are important targets of miR-31. These findings indicate that miR-31 inhibits metastasis, which suggests that it may be an ideal therapeutic target for breast cancer.

### 3.8. miRNA-342

The miR-342 was revealed to play a tumor suppressive role in regards to tamoxifen resistant breast cancer. Tumor resistance to tamoxifen, which is a selective estrogen receptor (ERα) modulator, remains a serious problem in clinical applications today, particularly in patients with carcinoma that overexpress the HER2 receptor tyrosine kinase. Although tamoxifen is one of the most common prescribed endocrine therapies, about 30%–40% of patients will fail adjuvant tamoxifen therapy and almost all of these patients with metastatic cancer will develop tamoxifen resistance as well [83]. Various studies have implicated HER2 as a significant factor in resistance to tamoxifen. Recent research found that there is an oncogenic splice isoform of HER2, HER2Δ16, which is also associated with metastatic breast cancer and resistance to endocrine therapy [84]. The miR-342 was revealed to contribute to tamoxifen resistance in several models, including cell lines that overexpress HER2Δ16 [85]. The miR-342 is downregulated in tamoxifen resistant breast cancer cell lines as well as in tamoxifen refractory breast cancers. Furthermore, the study also showed that miR-342 regulates gene expression in regards to tamoxifen-mediated apoptosis and proliferation. Reexpression of miR-342 may present a therapeutic approach in suppressing the growth of tamoxifen resistant breast cancer cells.

## 4. Oncogenic MicroRNAs

### 4.1. miRNA-10b

The miR-10b was one of the first miRNAs discovered to influence cancer metastasis. miR-10b is highly expressed in metastatic breast cancer cells and has a positive influence on cell migration and invasion *in vitro* [10,86]. It also initiates tumor invasion and metastasis *in vivo*. The metastatic MDA-MB-231 breast cancer cells were reported to express significantly higher levels of miR-10b in comparison to MCF-7 cells, which typically have little capacity to metastasize. Transduction of miR-10b into non-invasive SUM149 breast cancer cells resulted in increased size and invasiveness of tumors found in mice in comparison to tumors from nonmetastatic control SUM149 cells [10]. This study also emphasized the correlation between miR-10b expression levels in primary breast cancer and clinical progression in cancer treatment. Mechanistically, miR-10b was found to be activated by the transcription factor twist, which then causes an interruption of homeobox D10 (HOXD10) mRNA translation [87]. This results in increased expression of Ras homolog gene family member C (RhoC), which promotes cell invasion and metastasis [10].

Further research on the role of miR-10b in the metastasis of breast cancer showed that downregulation of miR-10b is one of the mechanisms by which breast cancer metastasis suppressor 1 (BRMS1) inhibits metastasis [88]. When BRMS1 was transfected into metastatic MDA-MB-231 and MDA-MB-435 breast cancer cells, the cell lines showed reduced metastasis [88]. The BRMS1 transfection also resulted in downregulated expression of miR-10b and RhoC, which suggests that miR-10b does affect the metastasis of breast cancer cells [89].

Recent exploration of miR-10b also demonstrated that treating cancer in mouse model with miR-10b antagonistic miRNAs (antagomirs) suppresses breast cancer metastasis [90]. Antagomirs, which are chemically engineered anti-sense RNA oligonucleotides against cognate miRNAs, are efficient and specific silencers of endogenous miRNAs. Silencing the miR-10b regulator resulted in decreased levels of miR-10b *in vivo* [3]. When mouse models with metastatic cancer were treated with miR-10b antagomirs, there was no resultant inhibition of primary tumor growth. Instead there were signs of decreased lung metastasis. These results suggest that miR-10b may be a good predictor of breast cancer metastasis because high levels are expressed in tumors that are more likely to metastasize into distant organs [91].

### 4.2. miRNA-21

The miR-21 has been found to be consistently overexpressed in many carcinomas, including breast cancer [92–94]. In accordance with these findings, further research revealed that miR-21 is also highly upregulated in breast cancer cell lines when compared to normal breast samples, which suggests that miR-21 may act as an oncogene [95]. Among the 157 human miRNAs that were analyzed by real-time RT-PCR arrays, miR-21 was considerably overexpressed in breast cancer tissues [93]. To further explore the role of miR-21 in breast cancer development and progression, the miRNA was suppressed in breast cancer samples by anti-miR-21, which inhibits cell growth *in vitro* and tumor growth *in vivo*. Suppression of miR-21 was found to be linked with reduced cell proliferation and increased apoptosis [96,97]. Another study also confirmed that miR-21 targets the tumor suppressor tropomyosin 1 (TPM1), and overexpressing TPM1 in breast cancer cells resulted in suppressed clonogenic potential [98]. Therefore, it can be concluded that the downregulation of TPM1 by miR-21 suppression may explain the inhibition of tumor invasion.

Additional analysis of metastatic MDA-MB-231 breast cancer cells demonstrated that suppressed miR-21 results in decreased invasion and lung metastasis [98]. This investigation also helped identify two more targets of miR-21, which are programmed cell death-4 (PDCD4) and maspin [99]. PCDD4 and maspin both negatively enhance invasion and metastasis and thereby provide more targets for miR-21 in human breast cancers. Another important target of miR-21 includes the tumor suppressor gene phosphatase and tensin homolog (PTEN) [100–102]. In a study involving 40 invasive ductal carcinoma samples from breast cancer patients, expression of miR-21 was revealed to be negatively correlated with expression of PTEN [103,104]. The inverse relationship between PTEN and miR-21 suggests that tissues with miR-21 expression represent an aggressive phenotype. However, other studies have also shown that although the upregulation of miR-21 correlates with invasive tumors, there is still no clear pattern observed for the target genes, such as PTEN and PDCD4 [102]. Further studies should explore miR-21 targets in order to appreciate the benefit of targeted therapy for breast cancer.

As mentioned above, miR-21 has been found to be overexpressed in breast tumors in comparison to normal breast tissue, and is also associated with advanced stage, lymph node positivity, and reduced survival time [45]. The role of miR-21 is not only important in its activity as a biomarker, but also as a functional target. A study in which MCF-7 breast cancer cells were transfected with anti-miR-21 oligonucleotides resulted in reduced *in vitro* cell growth and *in vivo* tumor growth in a xenograft mouse model [45]. The reduced cellular proliferation was associated with increased apoptosis and reduced levels of the BCL-2 anti-apoptotic protein. Overall, miR-21 has been shown to affect several targets, including PDCD4, TPM1, maspin, and PTEN. Studies on miR-21 suggest that miR-21 is an oncogenic miRNA and plays a role not only in tumor growth, but also in invasion and metastasis by targeting multiple anti-metastatic genes [7,105,106].

### 4.3. miRNA-155

The miR-155 is overexpressed in several human carcinomas, including breast cancer [92]. It is an effective suppressor of apoptosis, due to its effects on caspase 3, the most important caspase that is involved in the execution-phase of apoptosis [107]. Recent studies have investigated the targets of miR-155. The tumor-suppressor gene suppressor of cytokine signaling 1 (*SOCS1*) has been identified as a target of miR-155 in breast cancer cell lines [108,109]. The expression of *SOCS1* was found to be inversely correlated to miR-155 expression in human breast cancer cells. Ectopic expression of miR-155 also enhanced proliferation of breast cancer cells and the development of tumors *in vivo*. Furthermore, silencing of *SOCS1* in breast cancer cells reestablishes the oncogenic effects of miR-155, while restoring *SOCS1* expression promotes the pro-tumorigenesis function of miR-155, which indicates that miR-155 negatively regulates *SOCS1*.

Another study also showed that miR-155 is upregulated in normal mouse mammary gland epithelial cells (NMuMG cells) by the TGF-β pathway and it mediates TGF-β-induced epithelial-to-mesenchymal transition (EMT) and cell invasion [110]. Researchers have found that the ectopic expression of miR-155 in NMuMG cells disturbs proper tight junction formation between cell walls and promotes cell migration and invasion. Furthermore, miR-155 also directly inhibits the expression of *RhoA*, a gene that regulates various cellular processes, including cell adhesion, motility, and polarity. The miR-155-induced phenotypes were reestablished by expressing a miR-155 insensitive version of *RhoA* in miR-155 overexpressing cells. These results indicate that miR-155 is regulated by the TGF-β pathway and also downregulates the RhoA protein expression to enhance the acquisition of EMT phentype. Also, by demonstrating that miR-155 is highly expressed in invasive tumors but not in noninvasive cancer samples, it was shown that miR-155 is linked to cancer invasiveness in human breast cancers [45]. Therefore, the miR-155 appears to play an essential role in breast cancer metastasis due to its implications in the acquisition of EMT and increased potential for invasion and metastasis.

Another role of miR-155 in the regulation of cell survival is seen through the downregulation of its direct target FOXO3a in breast cancer. Ectopic expression of miR-155 induced cell survival and chemoresistance to several agents, while the knock-down of miR-155 resulted in apoptosis and increased chemosensitivity [111]. In this investigation, it was found that overexpression of miR-155 led to repressed FOXO3a protein with consistent mRNA levels, and the knock-down of miR-155 resulted in increased FOXO3a. This study revealed that there is an inverse correlation between miR-155 and FOXO3a in breast cancer cell lines, which suggests that miR-155 is an essential therapeutic target in breast cancer.

### 4.4. miRNA-373/520c

The miR-373 and miR-520c are prometastatic miRNAs [79,112]. In a genetic screen that overexpressed approximately 450 miRNAs in the nonmetastatic MCF-7 breast cancer cell line, authors found that miR-373 and miR-520c promoted migration and invasion *in vivo* and *in vitro*. Furthermore, several cancer lines even required miR-373 for migration and the miRNAs also elicited a migratory phenotype by inhibiting the expression of the metastasis repressor CD44 [113]. Ectopic overexpression of CD44 reduced migration of MCF-7 cells that express miR-373 and miR-520c, which indicates that the downregulation of CD44 is essential to the migration of these cells [114]. Finally, miR-373 was also shown to be upregulated while CD44 was decreased in metastatic breast cancer tissue specimens [115], which additionally implicate these miRNAs for their roles in breast cancer metastasis.

### 4.5. miRNA-375

Studies on miR-375 show conflicting results in both oncogenic and tumor suppressive roles. One particular study investigated miR-375 levels in estrogen receptor α (ERα)-positive breast cancer cell lines. Upregulation of ERα results in abnormal cell proliferation in the majority of breast cancers, but understanding of the mechanism behind this phenomenon is still unclear. Research showed that overexpression of miR-375 in ERα-positive breast cell lines contributed to proliferation [116]. In accordance, inhibiting miR-375 expression in ERα-positive MCF-7 breast cells resulted in reduced proliferation and ERα activation. The study also identified RASD1 as a potential target of miR-375. The miR-375 regulates RASD1 by targeting the 3′ untranslated region in RASD1 mRNA [116]. RASD1 contributes to cell proliferation by negatively regulating ERα expression. This suggests that there is a positive feed-forward loop, and that miR-375 plays an oncogenic role in breast cancer.

On the other hand, a conflicting study reveals that miR-375 may also have tumor suppressive role. Research on EMT and its role in initiating tumor cell invasion and metastasis showed that the acquisition to EMT exhibits resistance to a variety of cancer therapies, including tamoxifen [117]. To investigate the role of miRNAs in regards to resistance to tamoxifen, MCF-7 breast cells were exposed continually to tamoxifen to create a tamoxifen-resistant (TamR) model, which subsequently developed mesenchymal characteristics and increased invasiveness. Data showed that miR-375 was one of the top downregulated miRNAs in resistant cells and that the reexpression of miR-375 showed increased sensitivity of tamoxifen-resistant cells, and also partly reversed EMT phenotype [117]. The study also revealed that miR-375 directly targets metadherin (MTDH) and that there is an inverse correlation between the expression of miR-375 and MTDH in primary breast cancer cell lines. Furthermore, patients who were also exposed to tamoxifen were found to express higher levels of MTDH and experience shorter disease-free survival, and they also faced increased risk of relapse. Thus, concentrating on reexpression of miR-375 may serve as a potential therapeutic approach for breast cancer treatment.

### 4.6. miRNA-221 and miRNA-222

The miR-221 and miR-222 have both been identified as basal-like subtype-specific miRNAs, expressed in a basal-like subtype of breast cancer [118]. Both miRNAs function as regulators of EMT and the expression of these miRNAs results in increased cell migration and invasion. The miR-221 and miR-222 repress their target tricho-rhino-phalangeal syndrome type 1 protein (TRPS1), which in turn increases the EMT-promoting protein zinc finger E-box-binding homeobox 2 (ZEB2) [118]. Another study also demonstrated that miR-221 and miR-222 both directly target estrogen receptor alpha (ERα) and that overexpression of these miRNAs in breast cancer aid in the progression of the more aggressive basal-like breast cancer [119]. They repress proteins p27/Kip1 and p57, which are both cell cycle inhibitors, which results in increased proliferation [120]. These characteristics also aid in developing tamoxifen resistance in basal-type breast cancers. These findings suggest that both miRNAs play important roles in the promotion of clinically aggressive basal-like breast cancer.

Further insight revealed that miR-221 and miR-222 are differentially expressed and that both miRNAs are overexpressed in estrogen receptor negative (ER−) breast cancer [119]. Transfections of miR-221 and miR-222 synthetic mimetics into a nontransformed mammary cell line MCF10A resulted in induced EMT-like phenotypes, increased invasion and migration, and increased the levels of the mesenchymal marker vimentin. In addition, inhibiting miR-221 and miR-222 in the metastatic MDA-MB-231 breast cancer cell lines induced a reverse phenotype.

## 5. Other MicroRNAs

Although many miRNAs have been revealed to play significant roles in breast cancer progression or suppression, mechanistic roles or molecular functions are yet to be fully understood. However, increasingly rapid progress is being made on miRNA function in breast cancer metastasis. For example, it was found that miR-224 expression is considerably upregulated in breast cancer cell lines, and is particularly invasive in MDA-MB-231 breast cancer cells [121]. Furthermore, miR-224 inhibits the tumor suppressor Raf kinase inhibitor protein (RKIP) gene expression, which protects against metastasis and genomic instability. Metastasis is also induced by ectopic expression of miR-224 and reduced by its downregulation. These findings show that miR-224 may function as an oncogenic miRNA. Another potential oncogenic miRNA molecule is miR-135a, which has been implicated in several cancers, including breast cancer. It is highly expressed in metastatic breast cancer and found to be required for breast cancer migration and invasion [122]. The miR-135a targets HOXA10, which has been shown to induce p53 expression in breast cancer cells, and it is a metastasis suppressor in breast cancer [122]. The miR-135a suppresses the expression of HOXA10, which results in increased migration and invasion. In addition, miR-375 has been shown to be differentially expressed during breast lobular neoplasia, which promotes loss of mammary acinar polarity in *Homo sapiens* [123]. Research shows that invasive lobular carcinoma (ILC) of the breast leads to approximately 5%–15% of invasive breast cancers and maintains a clonal relationship with concurrent lobular carcinoma in situ (LCIS) lesions. It was revealed that miR-375 is upregulated during lobular neoplasia progression and overexpressed in ILC progression. When miR-375 was overexpressed in MCF-10A 3D culture model of mammary acinar morphogenesis, there was loss of cellular organization and progression of the hyperplastic phenotype. The transition from normal cells to invasive lobular carcinomas after upregulation of miR-375 suggests that it contributes to lobular neoplastic progression. Another pair of oncogenic gene regulators, miR-182 and miR-183, are overexpressed in ductal carcinoma *in situ* (DCIS) and lead to increased expression of chromobox homolog 7 (CBX7), DOK4, NMT2, and EGR1 [124]. A recent study revealed that suppressing miR-182 and miR-183 *in vitro* led to upregulation of their four target genes, which in turn resulted in upregulation of E-cadherin expression. This suggests that knocking out the two miRNAs may have important implications in reverting breast carcinomas to normal epithelial cell morphology.

Another study showed that miR-203 targets baculoviral IAP repeat-containing protein 5 (BIRC5) and Lim and SH3 domain protein 1 (LASP1) in human TNBC cells [125]. The miR-203 inhibits cell proliferation and migration in TNBC cells. In addition, upregulation of BIRC5 and LASP1 were able to reverse the effects of miR-203, which suggests that miR-203 may function as a tumor suppressor. Another miRNA that may function as a tumor suppressor is miR-30a. The miR-30a targets Vim, a gene that codes for vimentin, a protein that contributes to EMT phenotype [126]. Breast cancer cells exhibit low miR-30a level, and so it was demonstrated that overexpression of miR-30a leads to reduced migration and invasion of cancer cells. The findings of this study suggest that miR-30a may play an important role in inhibiting metastasis, which makes it an ideal therapeutic target for breast cancer treatment. In addition, another regulatory molecule with tumor suppressive characteristics is miR-7, which targets a widely upregulated signaling kinase, p21-activated kinase 1 (Pak1). Studies showed that miR-7 has an inverse correlation with Pak1 expression in human breast cancer cells [127]. miR-7 targets the 3′-untranslated region (UTR) of Pak1 and inhibits its expression. Furthermore, it is regulated by HoxD10, which is a homeodomain transcription factor that leads to increased cell invasiveness at low expression levels. Thus, during breast cancer progression, there are increased levels of Pak1 protein while miR-7 expression levels and HoxD10 are both downregulated as the cancer becomes more invasive. The study concluded that miR-7 inhibits the motility, invasiveness, and tumorigenic potential of highly invasive breast cancer cells. Moreover, evidence supports that miR-335 and miR-126 function as tumor suppressive miRNAs [78]. Both miR-335 and miR-126 expression are lost in most primary breast carcinomas of patients who relapse. miR-335 regulates a set of genes that collectively are associated with distal metastasis. Moreover, loss of expression of either miRNA results in low survival rates for breast cancer patients. miR-335 suppresses metastasis and migration by targeting the progenitor cell transcription factor *SOX4* and the extracellular matrix component tenascin C (*TNC*). SOX transcription factors have been shown to regulate progenitor cell development and migration. Thus, inhibition of miR-335 levels results in enhanced expression of *SOX4*. Moreover, miR-335 is restored, metastatic cell invasion is inhibited, and cells show reduced tumor growth and proliferation when miR-126 is restored. Since miR-335 and miR-125 are both downregulated across numerous highly metastatic cell lines and exhibit abilities to suppress metastasis of breast cancer cells, further exploration of either miRNA would be beneficial for breast cancer therapeutics research.

## 6. Conclusions

The miRNA-based breast cancer therapy is still relatively new, and emerging studies are only beginning to show the potential role of miRNAs in designing novel therapies for human malignancies, in general, and breast cancer, in particular. The miRNAs are ideal targets for breast cancer therapy because they are so intricately correlated with the progression of human breast cancer. Table 1 details the miRNAs discussed in this article, documenting the functional importance in defining the aggressiveness of breast cancer cells. Although there seems to be some contradictory reports and mechanistic explanations for certain miRNAs, research on these regulatory molecules has come a long way since their initial discovery. The miRNAs have transformed the modern understanding of cancer biology by analyzing tumor suppressive and oncogenic roles of miRNAs in various cancers. More research is emerging rapidly to detail further mechanistic understanding of these molecules. Recently, exploration of systemically delivered synthetic miRNA mimics, in conjunction with neutral lipid emulsion, has emerged as an effective potential cancer therapeutic [128]. Mimics of certain tumor suppressors, such as let-7 and miR-34a, which are both downregulated in lung cancer, were injected into mice models with non-small cell lung cancer (NSCLC) and resulted in reduced tumors. Successful systemic treatment of lung cancer patients with miRNA mimics could suggest possible treatment plans for breast cancer patients as well. However, a better understanding of the network of genes and cellular pathways regulated by miRNAs would certainly help improve our understanding of breast cancer pathogenesis, and thus will open newer avenues for improving the therapeutic outcome of breast cancer patients. A beneficial next step would be to identify the genome-wide targets of associated miRNAs. Furthermore, it would be interesting to translate our miRNA knowledge into a clinical setting to explore their practical applications of this new technology to modernized breast cancer treatment. Until that is possible, further research would certainly help in improving our understanding of the great potential that miRNAs may offer in cancer diagnosis, prognosis and potential target for the development of novel therapeutics.

## Figures and Tables

**Table 1:** miRNAs involved in breast cancer migration, invasion and metastasis.

miRNA	Potential Target(s)	References
**Tumor suppressor miRNAs**		

miR-125a	HER2, HER3, HuR	[51,54]
miR-125b	HER2, HER3, c-Raf	[54,55]
miR-200	BMI1, ZEB1, ZEB2, PLCG1	[34,35,43]
let-7	LIN28, HMGA2	[45,62]
miR-205	HER3	[70]
miR-206	ESR1	[76,77]
miR-34a	Bcl-2, SIRT1	[80]
miR-31	FZD3, ITGA5, M-RIP, MMP16, RDX, RhoA	[81]
miR-375	MTDH	[117]
miR-342	HER2Δ16	[85]
miR-203	BIRC5, LASP1	[125]
miR-30a	Vimentin	[126]
miR-7	Pak1	[127]
miR-335	SOX4, TNC	[78]
miR-126		[78]

**Oncogenic miRNAs**		

miR-10b	HOXD10	[10]
miR-21	BCL-2, TPM1, PDCD4, PTEN, MASPIN	[98–100,102]
miR-155	RhoA, SOCS1, Caspase 3, FOXO3a	[108,110,111]
miR-373	CD44	[115]
miR-520c	CD44	[115]
miR-375	RASD1	[116]
miR-221	TRPS1	[118]
miR-222	TRPS1	[118]
miR-224	RKIP	[121]
miR-135a	HOXA10	[122]
miR-375	*No Target Determined*	[123]
miR-182	CBS7, DOK4, NMT2, EGR1	[124]
miR-183	CBS7, DOK4, NMT2, EGR1	[124]

This table summarizes the reported roles of several tumor suppressor and oncogenic miRNAs. While most of the miRNAs are uniquely tumor suppressive or oncogenic, miR-375 has been reported as both, and is therefore listed in both the categories.
